# Self-Assessment of the Body and Social Competences in the Group of Mothers and Their Adult Daughters

**DOI:** 10.3390/ijerph16162824

**Published:** 2019-08-08

**Authors:** Bernadetta Izydorczyk, Katarzyna Sitnik-Warchulska, Kinga Ostrowska, Jolanta Starosta

**Affiliations:** Institute of Applied Psychology, Faculty of Management and Social Communication, Jagiellonian University in Krakow, Łojasiewicza 4, 30-348 Krakow, Poland

**Keywords:** self-assessment, body image, self-esteem, daughters, mothers, women, social competencies, assertiveness, intimate situations, social exposure

## Abstract

The main research objective of this study was seeking the predictive role of general self-esteem and the body image in social competences among women and their biological daughters. As it stands, there is a lack of research showing the mothers and their adult daughters at the same time in the context of measuring the same psychological variables, i.e., general self-esteem, self-assessment of the body and specific social competences in the scope of behaviour in intimate situations, situations requiring social exposure and assertiveness. The study group comprised 102 individuals; 51 pairs of mothers (40–64 years old, M = 51.33) and their biological daughters (19–25 years old, M = 22.49). The following instruments were used: The Rosenberg Self-esteem Scale, the Contour Drawing Rating Scale, the Body Esteem Scale, the Social Competence Scale, categorized interview (to measure BMI and collect data describing the criteria for selection to the research group). The significance of the differences and the stepwise regression analysis were performed. The results of the study demonstrated the following to be significant predictors of social competences in subjects: General self-esteem B = 0.615, discrepancy real-obligatory body image B = 0.275 among daughters, and physical condition B = 0.362 in mothers. The general self-esteem of daughters positively influences all verified types of their social competences (competences in intimate situations, in case of social exposure and ability to be assertive). However, it is the significant predictor only for mothers’ competences in dealing with situations of social exposure. Discrepancy real-obligatory body image: Seems to be the predictor of daughters’ social competences conditioning effectiveness in situations requiring assertiveness. The physical condition among mothers seems to be especially important for their assertiveness and effectiveness in intimate situations. The conflict between the real and the ideal body image is also an important aspect in predicting the assertiveness in the group of mothers. The study results can prove to be helpful in creating preventive and educational programs focused on self-esteem and social competencies in women, including the context of the relation between mothers and their daughters.

## 1. Introduction

The connections between the self-assessment of the body and social competences of mothers and their biological daughters is so far a very poorly studied research area. Although this issue seems to be important for developing scientific knowledge, which could support preventive healthcare and promote pro-health behaviours towards the body. Available sources of literature indicate that modern scientists often search for psychological and sociocultural factors influencing the development of the body image in the groups of healthy people and in groups of people presenting various types of eating disorders [1,2,3]. Moreover, scientific measurements of the relation between social competences and self-esteem, which include self-assessment of the body, have appeared for many years among the research topics conducted on adult population [4,5]. However, there are no studies that empirically document the measurement of dependence between the general self-esteem and self-assessment of the mother’s body and the general self-esteem and self-assessment of her adult daughter’s body. 

Analysis of research reports on the relationship between the general self-esteem and self-assessment of the body image indicates the important role of self-esteem in creating body shape dissatisfaction [6,7]. At the same time other research emphasizes the importance of self-assessment for the development of social relations and social skills [8,9]. As Harman, Hansen, Cochran and Lindsey [10] stated in their research, the level of self-esteem and social competences (alongside a higher level of social anxiety and aggression) affects the frequency of giving false information on the Internet. According to Garner’s research [11], possessed cognitive schemas concerning the pursuit of slimness and the need to achieve a slim figure have an impact on the level of self-esteem. Body weight, slimness and body shape became the basis for inference about the person’s own worth [11]. 

The importance of the influence of internalized norms of family environment and the emotional bond between the parents and the children on the body image are indicated by the concept of modelling by significant people [12] as well as by the psychoanalytic theories which emphasize the role of emotional bonds in shaping the body image [13]. Research indicates that parents, especially mothers, are the source of important information about the body image [14]. Information regarding the need to lose weight, which the individual receives from the mother, including those expressed indirectly or through modelling, is more significant for girls than boys [15,16]. The importance of family attitudes towards the body of adolescent children is also highlighted by cognitive-social theories. Parental attitudes (especially maternal) focused on the slimness of the body or taking care of the appearance may mediate the development of the disturbed body image among women [12]. The messages about the appearance are also significant [17]. The Tripartite Influence Model also indicates the importance of the mothers’ influence on the body image of their daughters [18].

The results of the research conducted by Domene, Socholotiuk and Young [19] show that in the process of entering adulthood: Mother-daughter relationship compared to mother-son relationship is characterized by fewer conflicts, greater involvement in joint projects and more open conversations, which may contribute to sharing content and patterns, and creating similarities between mothers and their daughters. The influence of messages received from a parent of the same sex on creating body-related problems in adolescence seem to be also confirmed by Izydorczyk [20] in a study conducted on a group of young patients suffering from anorexia and their mothers. 

Admittedly a few comparative studies and indications of the relationship between the body image of young women and its impact on social competences started appearing after 2016, but there are not many of them [21,22,23,24]. The conducted research so far most often included groups of girls and women without applying the criteria of lineal consanguinity between them. It is worth emphasizing that there is a lack of Polish research, in which pairs of related people would participate– mothers and their adult daughters at the same time in the context of measuring the same psychological variables, i.e., general self-esteem, self-assessment of the body and specific social competences in the scope of behaviour in intimate situations, situations requiring social exposure and assertiveness. 

## 2. Research Objectives and Questions

The authors of the presented article focused on examining the relation between the mother’s general self-esteem and the image of their body and general self-esteem and body image of their biological daughters. Considering the criterion of lineal consanguinity between mothers and daughters in the selection of the group the authors of this research referred to major and most significant assumptions of known psychological theories confirming the impact of emotional bonding and emotional adjustment of the parent to the child on the development of the body image in the course of development of the individual [13]. The body image of the adult daughter which was created under the influence of patterns of emotional relations between the child and the parent (for girls it is the pattern of relationship with their mother) is the psychological structure with significant importance in establishing social relationships and developing social skills for young women. 

On the other hand, to explain the relation between the general self-esteem and self-assessment of the body of mothers and the self-esteem of their adult daughters, the authors of the article adopted a cognitive model, which refers to the Higgins’ Self-Discrepancy theory [25]. This theory was applied by Thompson [26] to explain the psychological mechanisms of body image development with an indication of special significance of the discrepancy in the assessment of the body image between the actual self (real body appearance), ideal self (evaluation of desirable body image) and ought self (how body image should look in relation to social requirements). As the research results indicate, it is often not the actual appearance, but the personal satisfaction with it that regulates functioning of the individual, including social competences [27,28]. In context of the aforementioned studies, it seems that this satisfaction is regulated through the relationship between the adolescent daughter and her mother, both in context of importance of the mother’s assessment and observation of her in relation to her own body. 

The lack of eating disorders and other psychiatric disorders (with symptomatology of body image disorders) were considered a criterion in the process of selecting pairs of mothers and their biological daughters to the research group. It was decided to study adult daughters and their mothers. Young adulthood is a time associated with a great need of social activity and making social relations which can be helpful in their future professional work. It is a time of life associated with the search for a partner and the beginning of stabilization in the relationships. In aforementioned social situations, the body image which is attractive to the others and to the person itself becomes an important factor in shaping self-assessment. It was assumed that examined women (mothers and daughters) have already shaped the personality structure and body image (according to Higgins’ theory: The assessment what I am, how I would like to be/look, how I should look). Due to the above it can be assumed that the influence of the educational and family environments have already been internalized. Consequently, it can be presumed that each of the examined mother and daughter have shaped the personality which will be characterized by stability. Therefore, it can be assumed that adult daughters, who do not reveal mental disorders and eating disorders, will show independence from the influence of their mother’s attitude towards their own body. Moreover, it can be suspected that daughters will use social competences which enable them to maintain health and to function properly in the social environment. 

Considering the field of the study it was worth noting whether and in what way already established general self-esteem and self-assessment of the body image in adult daughters are predictors of developing social skills for these young women. These social competences determine their effectiveness of coping with intimate situations in close relationships, social exposure and with situations that require assertiveness (setting boundaries, the ability to express his/her own opinion without an inadequate feeling of fear or guilt). Moreover, in the study it was examined to what extent the general self-esteem and self-assessment of the mother’s body is a predictor of their social skills. At the end it was checked whether mothers and their adult daughters differ in terms of strength of influence of general self-esteem and body self-assessment on their social competences. 

Research questions:

(1) Is the general mothers’ self-assessment different from the daughters’ general self-assessment? 

(2) Are there differences between social competences of mothers and social competences of their adult daughters?

(3) To what extent the self-assessment of body among mothers and their adult daughters explain their social skills in intimate situations, social exposure and situations requiring assertiveness?

(4) To what extent the general self-assessment among mothers and their adult daughters explain their social skills in intimate situations, social exposure and situations requiring assertiveness?

## 3. Material and Methods

### 3.1. Procedure

The research was conducted in 2017 in the urban environment in which the examined women lived, studied or worked. 

On the basis of literary sources the dependent variable “social competences” was defined as complex skills of a person, which determine the effectiveness of dealing with such situations as: Intimate situations (variable describing the degree of emotional and physical satisfaction with intimate and physical contact with another person), social exposure (share someone’s opinion without fear) and situation requiring assertiveness (setting boundaries). In the model of our own studies, an additional variable was also distinguished: Body mass index, BMI. 

First, the independent variable was general self-esteem, which was defined as a relatively permanent disposition of the person to evaluate itself in a positive or negative way [29,30]. Second, the independent variable was self-assessment of the body defined for the needs of the study as a set of content describing: The power of discrepancy between the real and desired body appearance, the level of feeling of own sexual attractiveness, physical condition and the level of control of their body weight among mothers and their adult daughters. 

### 3.2. Characteristics of the Study Group

The study group consisted of 102 women—51 pairs of mothers (40–64 years old; M = 51.33, SD = 5.31) and their biological daughters (19–25 years old; M = 22,49, SD = 1,65). Such research group provides the basis for conducting appropriate statistical analyses to answer the research questions. 

The selection criteria for the research group included: Age of the participants (19–64 years old), gender (adult women in the period of late adolescence, early, middle and late adulthood), the marital status of the mother with an adolescent or young adult daughter, body mass index (BMI values 18.5–24.9, in the norm for a given age), lack of eating disorders (anorexia nervosa, bulimia and other mental disorder), lack of presence of diseases requiring hospitalization or chronic treatment at the moment of the research, lack of disability and physical deformation of the body. The semi-structured clinical interview was used to collect these data. The interviews were conducted by one of the authors. The research group was homogeneous in terms of socio-demographic conditions (place of residence, age, gender). Examined individuals lived in southern Poland. 

Group I consisted of 51 Polish women, students of humane, social and biological sciences, coming from conjugal families or from families where their parents were divorced but both of them participated actively in the upbringing of the examined daughters. Women lived with parents or alone due to their studies. They were maidens and they did not have children (BMI = 21.0). Group II was formed by 51 Polish women, mothers of the examined daughters (BMI = 24.0). This group included both working and unemployed women. 

### 3.3. Instruments

To measure general self-esteem the Rosenberg Self-esteem Scale was used, in the polish adaptation by Dzwonkowska, Lachowicz-Tabaczek, Łaguna [31]. Reliability: Cronbach’s *alpha* 0.81–0.83. The indicator of the variable is the total score of 10 items of the SES scale. 

Two tests were used to measure mothers’ and daughters’ body self-assessment:

- The Contour Drawing Rating Scale by Thompson and Gray [32]. The research procedure consists of presenting nine female body shapes from which the research subject should choose three body figures which correspond to her current body shape, her ideal figure and the one she thinks a woman should own. The indicator of the body self-assessment is the numerical value describing the level of discrepancy between the assessment of the person’s current (real) body shape, the figure she would like to possess (ideal body image) and the body shape women should have (an obligatory body image). Reliability: Cronbach’s *alpha* 0.69–0.84.

- The Body Esteem Scale (BES) in the polish version developed by Lipowska and Lipowski [33] —total value of raw results in scales: AS—sexual attractiveness, PC—physical condition, WC—weight control. BES contains 35 test items. The test reliability is considered satisfactory (Cronbach’s *alpha* 0.80–0.89). Factor—sexual attractiveness allows describing the attitude of the subject to those parts of the body which cannot be changed by physical exercises (e.g., assessment of the satisfaction of the appearance of the lips or breasts). They can be emphasized only through the use of cosmetic treatments. Factor-weight control—describes the level of control of behaviours related to body weight, nutrition and the level of focus on the food. Due to this factor the body parts which can be changed by physical activity are important. Factor-physical condition—characterizes physical attributes of the person such as endurance, strength and agility [33]. 

To measure the variable-social competences the Social Competence Scale (KKS) by Matczak [34] was used. Reliability ratio—Cronbach’s *alpha* 0.74–0.94 (for women). Scale measuring social skills in the area of intimate situations (15 items), in terms of coping with situations of social exposure (18 items) and competences in the situations requiring assertiveness (17 items). The indicator of social skills of the respondent is the total raw score in all scales and partial results achieved in three scales (intimate situations, social exposure and assertive situations). 

To measure BMI and collect data describing the criteria for selection to the research group the categorized interview was used. The tool included questions about current body weight and height (18.5—underweight, 18.5–24.99—normal weight, ≥ 25.0—overweight), place of residence, gender, marital status of mothers which have a daughter in the age of late adolescence or young adulthood (19–25 years old), lack of eating disorders (anorexia nervosa, bulimia and other mental disorders), lack of presence of diseases requiring hospitalization or chronic treatment at the moment of the research, lack of disability and physical deformation of the body. 

### 3.4. Ethical Approval

Ethical approval was obtained from relevant institutional ethical review committees and the research conducted in accordance with national and international regulations and guidelines. The written informed consent was obtained from all participants. 

### 3.5. Statistical Methods

The study results were statistically analysed using the IBM SPSS Statistics (Statistical Package for the Social Sciences) software. The significance of the differences between the mean values of the severity of general self-esteem indicators, body self-assessment and social competences in group of mothers and daughters was measured in the first stage of statistical analysis. In the second stage, the stepwise regression analysis was performed. An attempt was made to estimate the strength and direction of an impact of possible predictors of social competences. Due to the large number of independent variables the forward selection was applied by subsequently including relevant predictors into the model. The process lasted until the appearance of the first predictors whose level of significance exceeded the permissible values of *p* < 0.05. In that way the models were cleaned from unnecessary and weak predictors. The obtained models have satisfactory prediction [35]. 

As an acceptance or rejection criterion for the proposed hypotheses, the level of significance was set at *p* = 0.05.

## 4. Results

At the first stage of analysis statistical differences of examined variables were estimated. The results of the analysis are presented in Table 1. 

Statistical analysis of mean values indicate that the general self-esteem of mothers and their adult daughters is significantly different. Daughters are characterized by lower level of general self-esteem than their mothers. Furthermore, an analysis of the differences between mothers’ and daughters’ social competences in the scope of the way of dealing with social exposure situations was also performed. The results showed that daughters have better competences in a situation of social exposure than their mothers. In the case of other variables, both self-assessment of the body and other social competence factors proved to be not significantly different between the two groups. A similar situation was observed in the case of discrepancies between the real and ideal body self-assessment and also between the real and obligatory body image. The variable also did not differentiate mothers and their adult daughter. Both research groups experience similar conflict between the real body image and the assessment how women body should look like. In the case of the level of severity of other factors which comprise the self-assessment of the body (sexual attractiveness, physical condition, weight control), there is no difference between the group of mothers and a group of daughters. Differences in the level of social competences were also proved statistically insignificant in the field of such factors as: Effectiveness in situations requiring assertiveness and in intimate situations. 

To find answers to the third and fourth research question a stepwise regression was used. It allowed to identify predictors, which significantly affect the level of social competences specific to mothers and their daughters. On the basis of the adjusted R^2^ it can be concluded that the degree of explained variance of the dependent variable-social competences ranges from 11 % to 46 %. Although all regressions were statistically significant (*p* < 0.05), the values of R^2^ coefficients clearly suggest that the dependent variable may be influenced by other variables which were not included in the presented study. The results in Table 2 present the self-assessment of the body, which significantly explain the social competences analysed in the study in groups of mothers and their adult daughters. 

The stepwise analysis and obtained values of *Beta* coefficients indicate that general self-esteem is a significant independent variable for three factors of social competences in the group of daughters. Furthermore, it is also significant in explaining social competences in situations of social exposure in a group of mothers. The general self-esteem of daughters positively influences on all verified types of their social competences (competences in intimate situations, in case of social exposure and ability to be assertive). This means that a higher level of the daughters’ general self-esteem is associated with having better social competences in indicated areas. On the other hand, general self-esteem is a significant predictor only for mothers’ competences in dealing with situations of social exposure. The dependence proved to be positive, which means that the higher the mothers’ general self-esteem, the more effective they cope with situations of social exposure. 

Statistical analysis confirmed that the variables included in the self-assessment of the body are variously explained by different predictors in the group of mothers and in the group of their daughters. Predictors regarding social competences considered as generalized skills are different depending on the research group. Within the group of mothers this predictor is the physical condition. The better their physical condition, the greater are their general social competences. On the other hand, within the group of daughters, the predictor of the response variable was the discrepancy between the real and obligatory body image. The bigger the discrepancy between the daughters’ assessment of the real body figure and their assessment of how their body should look like, the higher the general social competences they have (positive effect). 

Furthermore, the predictor of social competences describing the effectiveness of behaviour in intimate situations is also different depending on the research group. Self-esteem is the predictor of social competences in intimate situations (emotional and physical satisfaction from the physical/intimate contact) within the group of daughters. On the other hand, physical condition is the predictor of social competences in the group of mothers. All predictors indicate a significant positive impact. The divergence of predictors in the explanation of self-assessment of the body indicates differences in the identified explanatory variables. Therefore, the examined mother builds their own competences in dealing with intimate situations by focusing on their own vitality and health. In contrast, general self-esteem is the only factor affecting the behaviour in intimate situations in the group of daughters. Self-assessment of the body does not have a significant meaning in how adult daughters deal with intimate situations. 

Indicators of self-assessment of the body proved to be predictors of social competences conditioning effectiveness in situations requiring assertiveness in both research groups. It is important to mention that these are various predictors, they describe different aspects of assessment of the body. The discrepancy between the ideal and real body image and physical condition are the predictors of the mothers’ social competences. Once again the assessment of one’s vitality and health has a significant impact on the mother’s assertive behaviours. However, in the group of their adult daughters, the predictor of social competences is discrepancy between their real and obligatory body image. It should also be noted that general self-esteem is also a significant predictor of the mother’s assertive behaviours. All predictors have a significant positive impact. 

The obtained results indicate that general self-assessment is the predictor of social competences which determine effectiveness in situations of social exposure in both research groups. This is the only such a case in the study. The results implicate the positive effect, which means that the higher is the self-esteem among mothers and their adult daughters, the greater are their social competences. The other components of variables included in the research model—general self-esteem and self-assessment of the body, proved to be irrelevant in explaining the mothers’ and daughters’ social competences in situations of social exposure.

## 5. Discussion

Statistical analysis show a significantly lower general self-esteem and higher social competences in situations of social exposure in the group of daughters. On the other hand, mothers have a significantly higher general self-esteem and lower social competences in the case of social exposure. Perhaps this effect is the result of the dynamics of changes in the development in subsequent stages of life. Robins et al. [36] indicate the changes in value of the general self-esteem. They analysed the result of surveys obtained from more than three hundred people aged from 9 to 90. They observed a tendency to possess a lower self-esteem during the adolescence than in the childhood. Self-esteem tends to increase in adulthood and decrease in the elderly. It is worth mentioning that in the presented study both groups—mothers and daughters were adults. However, daughters were at the stage of young adulthood or late adolescence. None of the examined daughters has been a parent yet. Self-assessment significantly fluctuates during adolescence and it is generally lower for women than for men during this period of life [36,37]. These differences seem to disappear over the age of 30. Self-esteem for women in the period of adolescence and young adulthood seems to be more associated with how physical attractiveness is promoted in media coverage [1,2,37]. On the other hand, Robins et al. referred to the known developmental theory which indicated that full adulthood is associated with acquisition of psychological maturity and redirecting attention to self-improvement and being productive. One wonders whether this type of maturity, and thus greater stability, is not related to the decrease of the need to obtain social confirmation in this stage of life. This could explain the lower results obtained by mothers in the scale of social competences in situations of social exposure. However, this issue requires further research.

It is worth mentioning that in the remaining categories of social competences and respective components of the body image, no statistically significant differences between the group of mothers and their daughters were found. This can confirm the fact that there are similar patterns of self-image in the mother-daughter relation, which was indicated by Domene et al. [19]. Arroro et al. conducted studies on a population of 286 family triads (mothers, daughters, siblings). They measured, among other issues, social competences and the mothers’ and daughters’ (over 21 years old) body image. The obtained results confirmed the significant impact of parents’ attitudes on their children’s self-image and self-assessment of their body. The source material indicates the importance of the mothers’ attitude towards their own body and how it can influence the development of the sense of identity and self-worth of their daughters [22,23]. 

The regression analysis revealed very interesting results for both research groups. Predictors for the variable—social competences were different in the group of mothers and daughters. Examined daughters seems to be more motivated to develop their social competences, both general and in situations which requires assertiveness if they focus more on striving to achieve the body shape, which was enforced by society standards of beauty. The predictive importance of factor—the discrepancy between the assessment of the real body image and the body figure that woman should have (obligatory body assessment) for development of social skills was indicated by Johns and Peters [38]. These authors emphasized that the discrepancy between the assessment of the real body image (how someone’s body really look like in their eyes) and the assessment in the scope of ought self (how someone’s body should look like in the eyes of others) increases the fear of establishing social relations. It may be related to the current socio-cultural standards and the pursuit of achieving them [1,2].

On the other hand, beyond the self-esteem, physical condition was the main predictor of general social competences and also skills related to assertiveness and dealing with intimate situations in the group of mothers. Taking care of the person’s own body figure seems to be particularly important above the age of 50, which can be related to a slower metabolism characteristic for that age. Middle aged women usually already experienced a number of important events in their lifetime, such as marriage, birth and growth of children, development of the career and achieving of professional goals, empty nest syndrome or changes in organism, which lead to menopause. Taking developmental challenges too early or too late or focusing on the biological clock can have a big influence on the person’s self-esteem in their middle age [39]. Lachman [40] in his study showed that changes in the physical and mental condition caused by ageing and decrease of health are considered to be the worst aspects of midlife. However, he emphasizes that despite the fact that it is the time of crises and loss (including those related to person’s own biological changes), it is also the time of taking new initiatives and self-development. Undoubtedly, it is also a time of retransforming the parental relationship into the partner relationship after the children leave the house and start living on their own. Physical closeness and satisfaction with intimacy require energy and physical agility. 

It is worth mentioning that Widerman’s [41,42] research indicated that the dissatisfaction with oneself and the body is connected with the difficulties in experiencing the intimate situation. In turn, Early, Ashmore, Mahkijani and Longo [43] showed in their research that people who present an attractive appearance are perceived as smarter, warmer, happier, more interesting and successful, with better social position and higher social skills. This could indicate that examined mothers pursue an effective relationship with others by focusing on their physical resources. It cannot be excluded that the obtained results are conditioned by the life balance characteristic for this period of life. However, this issue requires further research. 

### Limitations and Future Directions

The presented research has the character of cross-sectional studies focused on the intergroup differences and relationship between the variables. The research aimed at studying the relation between individual variables in mothers and their biological daughters, which would be valuable in the future. Valuable information may also be provided by longitudinal studies, which draw attention to the stability of the examined variables and study the relationship between the variables over time. Moreover, studies including the relationship/kinship between the mother and daughters of different age would also be valuable. Certainly there are also other variables conditioning social competences in the group of mothers and their daughters. However, the self-assessment of the body is little known as a predictor in this aspect. According to the authors of this article, the presented limitations do not negate the novelty of the obtained results on the importance of the self-assessment of the body in the diversification of social competences in the group of mothers and their biological daughters. 

## 6. Conclusions

The analysis of the data verifies the research question asked by the authors. The following conclusions can be made on the basis of the obtained results:

(1) Self-esteem is an important predictor of the mothers’ and their biological daughters’ social competences.
(2) The obligatory beliefs about how the women body should look like, its assessment and self-assessment of the body prove not to differentiate the mothers from their biological daughters. The similarity in this aspect may be the result of kinship and associated with biological and psychosocial processes (i.e., upbringing, modelling, internalization, transmission).
(3) The observed differences in the level of general self-esteem (lower level in the group of daughters) and social competences in the field of dealing with social exposure (higher level in the group of daughters) may be the result of developmental changes related to growth in subsequent stages of life.
(4) Physical condition proves to be a particularly significant predictor of social competences of mothers at the age of 50. It seems to be especially important for assertiveness and effectiveness in intimate situations. However, tension and conflict between the real and obligatory body image is the most important aspect in predicting the social competences in the group of adult daughters. It may be related to attaching importance to pursue social expectation in this period of life.

In order to strengthen social competences of young women, it is important to consider self-assessment of the body. It is also significant to include the context of the body image and social competences presented by their mothers, who raised them.

## Figures and Tables

**Table 1:** Analysis of examined variables by the t-student test in the group of mothers and daughters (N = 102).

Variables	Group I (Adult Daughters) (n = 51)		Group II (Mothers) (n = 51)		Student’s T-distribution *t*(50)	Significance (*p*)
	*M*	*SD*	*M*	*SD*		
General self-esteem	28.98	4.72	30.16	3.79	−1.35	*
**Body self-assessment**						
Sexual attractiveness	45.43	6.71	47.22	8.22	−1.48	in
Weight control	30.43	7.75	32.59	10.89	−1.17	in
Physical condition	30.02	5.86	31.16	6.87	−1.09	in
The discrepancy between the real and the ideal body figure	1.90	1.33	1.84	1.24	0.22	in
The discrepancy between the real body shape and the one a woman should have	1.73	1.33	2.22	1.38	−1.85	in
**Social competences**						
Social competences (overall score)	172.28	22.70	168.00	20.83	0.97	in
Social competences conditioning effectiveness in intimate situations	44.53	6.03	42.61	5.79	1.51	in
Social competences conditioning effectiveness in situations of social exposure	52.53	8.49	49.28	8.61	2.21	*
Social competences conditioning effectiveness in situations requiring assertiveness	44.25	7.85	44.78	6.39	−0.36	in

In-insignificant; * *p* < 0.05.

**Table 2:** Predictors which explain the level of social competences and their components in the group of mothers and their adult daughters.

Dependent Variable	Independent Variables	
	Daughters (N = 51)	Mothers (N = 51)
Social competences—(total score)	R^2^ = 0.410; F(3,47) = 10.90; *p* < 0.001 Predictors: Genera self-esteem B = 0.615 *** Discrepancy real-obligatory body image: B = 0.275 *	R^2^ = 0.205; F(3,47) = 4.04; *p* < 0.01 Predictors: Physical condition B = 0.362 *
Social competences conditioning effectiveness in intimate situations	R^2^ = 0.318; F(2,48) = 11.23; *p* < 0.001 Predictors: General self-esteem B = 0.632 ***	R^2^ = 0.247; F(1,49) = 16.31; *p* < 0.001 Predictors: Physical condition B = 0.497 ***
Social competences conditioning effectiveness in situations of social exposure	R^2^ = 0.225; F(3,47) = 4.57; *p* < 0.01 Predictors: General self-esteem B = 0.439 **	R^2^ = 0.113; F(2,48) = 3.05; *p* < 0.05 Predictors: General self-esteem B = 0.326 *
Social competences conditioning effectiveness in situations requiring assertiveness	R^2^ = 0.461; F(4,46) = 9.85; *p* < 0.001 Predictors: General self-esteem B = 0.567 *** Discrepancy real-obligatory body image: B = 0.680 ***	R^2^ = 0,151; F(2,48) = 4,27; *p* < 0.01 Predictors: Physical condition B = 0.402 ** Discrepancy real-ideal body image: B = 0.364 *

In-insignificant * *p* < 0.05 ** *p* < 0,001 *** *p* < 0.001.
